# A role for viral infections in Parkinson’s etiology?

**DOI:** 10.1042/NS20170166

**Published:** 2018-04-16

**Authors:** Laura K. Olsen, Eilis Dowd, Declan P. McKernan

**Affiliations:** Pharmacology and Therapeutics, National University of Ireland, Galway, Ireland

**Keywords:** neurodegeneration, neuroinflammation, Parkinsons disease, viral infection

## Abstract

Despite over 200 years since its first description by James Parkinson, the cause(s) of most cases of Parkinson’s disease (PD) are yet to be elucidated. The disparity between the current understanding of PD symptomology and pathology has led to numerous symptomatic therapies, but no strategy for prevention or disease cure. An association between certain viral infections and neurodegenerative diseases has been recognized, but largely ignored or dismissed as controversial, for decades. Recent epidemiological studies have renewed scientific interest in investigating microbial interactions with the central nervous system (CNS). This review examines past and current clinical findings and overviews the potential molecular implications of viruses in PD pathology.

## Introduction

Parkinson’s disease (PD), the most common neurodegenerative motor disorder, is generally characterized by the selective degeneration of dopaminergic neurones in the substantia nigra pars compacta (SN) of the midbrain, resulting in decreased dopamine (DA) transmission throughout the nigrostriatal pathway [1]. PD symptoms include resting tremors, unstable posture, bradykinesia, rigidity, and non-motor symptoms (such as dysphagia, olfactory impairment, sleep disturbances, dementia, and constipation) [2–4]. This progressive neurodegenerative disease can be familial (associated with early onset) or sporadic [5–8]. Hallmark pathological features of both familial and sporadic PD include uncontrolled protein aggregation (primarily α-synuclein fibrils forming Lewy bodies), oxidative stress (OS), mitochondrial dysfunction, chronic neuroinflammation (including microglia activation and astrogliosis), and autophagy disruption [5,9–11].

Despite over two centuries of investigation, the cause(s) of most cases of PD are still unknown. Epidemiological studies suggest there is a gene–environment interaction involved in the development of sporadic/idiopathic PD (iPD). Previous studies suggest a correlation between iPD development and exposure to pesticides or heavy metals, traumatic brain injury, and viral/bacterial infection [12–14]. Although multiple reviews describe the association between pesticides and PD (most notable rotenone and paraquat) [15,16], there are few reviews detailing all the epidemiological, *post-mortem*, and preclinical evidence surrounding the association between viral infections and PD. As urgently expressed by both scientists and clinicians in a recent article by Itzhaki et al. [17], previous studies identifying microbial associations with Alzheimer’s disease (AD) (the most common neurodegenerative disorder) [18,19,20,21,22] have largely been ignored or dismissed as controversial despite the current lack of progress for understanding or curing this disease. Similarly, the same lack of progress and dismissal of previous epidemiological findings regarding viral infections can be said for PD. The purpose of this review is to revisit historical conclusions, provide a comprehensive update on recent clinical findings, and overview the potential molecular and cellular implications of neurotropic viruses in PD pathology.

## Viruses as a risk factor for PD: sifting through historical and clinical evidence

The first suggestion of a relationship between viral infections and PD was the 1920–1930s influenza epidemic, which was associated with encephalitis lethargica (EL) [23]. Although EL patients exhibited drastic irregularities in disease progression and displayed ‘symptomatological polymorphisms,’ EL has been described as a type of ‘sleeping sickness’ which can include headache, nausea, fever, uncontrollable sleepiness, catatonia, and sometimes coma [24]. The EL epidemic coincided with an equally significant influenza pandemic (the Spanish influenza), leading many clinicians and other prominent scientists from that time to believe there was a causal relationship (or at least an epidemiological association) between these conditions [25]. Multiple studies investigating the preserved brain samples of EL patients from the epidemic years (1918–1930) found no evidence of the 1918 influenza virus in these tissues [26–28]. Also, 1918 influenza-derived sequences revealed mutations in two surface protein-encoding genes that suggest this viral strain was incapable of replicating outside of the respiratory system [29,30]. Reports from more current cases of EL suggest that EL may be an auto-antibody disorder [23,31–37].

Since the EL epidemic, numerous cases of post-encephalitic Parkinsonism (PEP) after certain viral infections (H5N1, coxsackie virus, Japanese encephalitis B., St. Louis viral encephalopathy, and HIV) have been reported, but these cases of Parkinsonism often do not exhibit the same cellular or molecular pathologies as seen in PD and are suggested to be ‘phenocopies’ of PD [38–44]. Although these acute cases of viral infection did not present with classical PD, these findings (along with the believed EL association with PD) led to multiple clinical studies in the late 1970s and early 1980s investigating the relationship between viral infections and PD.

Studies examining the relationship between viruses and PD are detailed in Table 1. A study by Elizan et al. [45] found a significant relationship between viruses (herpes simplex virus (HSV), measles, and influenza A) and iPD, but these findings may be confounded since their control group included amyotrophic lateral sclerosis and AD patients (conditions which have since been suggested to be associated with certain viral infections themselves). Another set of studies found an increased incidence of PD amongst those chronically infected with hepatitis C virus (HCV), but these studies are confounded by the fact that some HCV patients received interferon (IFN) treatments; with a follow-up investigation finding a much stronger relationship between IFN-treated groups and PD (249 PD incidents/100000 person-years) than non-IFN-treated groups (30 PD incidents/100000 person-years) [46–48]. Multiple studies by Marttila and colleagues, using a variety of antibody detection techniques (complement fixation, RIA, indirect immunofluorescent assay, microindirect hemagglutination), found a significant increase in HSV antibody titers and mean HSV titer in iPD patient serum [49–51]. A study using the microindirect hemagglutination test was able to differentiate between HSV-I and HSV-II; finding that increases in antibody titers and mean titer amongst iPD patients was specific to HSV-I only (not HSV-II) [51]. Other studies have questioned iPD patients for their history of HSV, measles, and influenza A infection with conflicting results (see Table 1). Although a significant association was found between severity and frequency of influenza A infection and PD incidence (with no association for HSV), the conclusions based on these studies are limited in that they rely on accurate patient memory and interpretation of their condition (patients cannot be expected to correctly diagnose their previous exposure or infection with viruses) [52,53]. A more recent study that examined PD patient serum found a more frequent incidence of HSV-I infections amongst iPD patients [54], further supporting the findings of the Marttila studies. Based on the aforementioned clinical evidence, HSV-I and strains of influenza A will be reviewed for their neurovirulence and association with PD-like pathology in the central nervous system (CNS). Molecular and cellular events associated with HSV-I/influenza A infection will be discussed for their potential implications in PD.

**Table 1:** Viral infection associations with PD

Viral infection	Relation to PD	Study
**HSV**	↑ incidence (HSV-I)	[49–51,55]
-Enveloped, linear dsDNA genome (152–154 kb)	↑ incidence	[53]
-Causes blistering of mouth/genitals, latency in neurones	↑ incidence (HSV-I)	[54]
	No association	[52]
**Influenza virus A**	↑ incidence	[53]
-Enveloped, linear ssRNA(–) genome (13.5 kb)	↑ incidence	[52]
-Causes fever, cough, runny nose, malaise	No association	[49]
**Measles virus**	↓ incidence	[52]
-Enveloped, linear ssRNA(–) genome (15–16 kb)	No association	[53]
-Causes rash, white spots, cough, red eyes	No association	[55]
**Cytomegalovirus**	↓ incidence	[52]
-Enveloped, linear dsDNA genome (~200 kb)	No association	[51]
-May cause mononucleosis, pnuemonia	No association	[54]
**Coronavirus**	No association	[56]
-Enveloped, linear ssRNA(+) genome (27–32 kb)		
-Causes upper/lower respiratory infection, sometimes gastroenteritis		
**Mumps**	↑ incidence	[53]
-Enveloped, linear ssRNA(–) genome (15 kb)		
-Causes parotid gland swelling, malaise		

## CNS viral entry: HSV-I and influenza A

Since the primary disease pathology characteristics of PD exist in the CNS, it may be relevant (but possibly not crucial) to study the neurovirulence of viruses associated with PD. HSV-I and influenza A have very different life cycles, resulting in different strategies for survival/replication within the host. Influenza A is generally a transient infection, lasting only a few weeks inside the host [57]. On the other hand, an acute infection of HSV-I (presented as epithelial blistering in the mouth or genitalia) is followed by viral latency, which is generally established in the trigeminal ganglia (TG) [58]. Although dormant, HSV-I is a chronic infection that maintains latency in sensory ganglia that innervate the brainstem and cerebellum of the CNS [59,60]. Primarily residing in the respiratory system during acute infection, influenza A can enter the CNS through the olfactory nerve [61]. Found to be axonally transported via cytoskeleton intermediate filaments, influenza A can follow olfactory neurone projections through the cribiform plate in the nasal cavity into the olfactory bulbs and olfactory tracts of the CNS [62,63].

Previously, viral entry of either HSV-I or influenza A into the CNS was considered fatal (or nearly fatal) via the rare condition of herpes simplex encephalitis (HSE) or acute encephalitis, respectively [38,64,65]. More recent findings now suggest that viral entry into the CNS does not necessarily result in a drastic, usually fatal, immune response. Although many studies have not found the existence of HSV-I DNA or antigens in *post-mortem* PD patient brains [66,67]; multiple studies have found HSV-I DNA in the brains of normal aged humans and AD patients [22,66,68–70]. The presence of HSV-I DNA was associated with increased age and the characteristic amyloid-β plaques found in AD [70,22]. Determining the neurovirulence and brain cell localization of influenza A in the CNS of PD patients is far more difficult since it is a transient infection. Although partially determined by the route of infection, preclinical mouse models of neurovirulent strains of influenza A have found this infection to successfully enter the CNS and localize in the SN, thalamus, hippocampus, locus coeruleus, ganglia (trigeminal, vagal, spinal, and sympathetic trunk ganglia), olfactory bulbs, and thoracic spinal cord around day 10 post-infection [39,71–73]. Influenza A antigens were also found to preferentially exist in catecholaminergic neurones, meninges, and ependymal areas [74]. Despite influenza A entry into the CNS in these mouse models, viral replication and maintenance in the CNS did not extend past 2 weeks, and was generally non-existent in the CNS by day 21 post-infection. More relevant to PD pathology, the neurotropic H5N1 influenza virus was found to induce long lasting microglia activation and α-synuclein phosphorylation and aggregation in the mouse SN post-infection [39,75].

An increased incidence of HSV-I DNA in the CNS and increased sensitivity to respiratory infections amongst the elderly [76,77] is worth noting since the greatest risk factor for PD is old age [78]. With age, the blood–brain barrier (BBB) becomes more permeable, resulting in more fluid entry of peripheral proteins into the CNS (including neurotoxic peripheral pro-inflammatory mediators) [79]. The immune system is also compromised in the elderly, with increases in pro-inflammatory cytokines and decreases in lymphocytes [80]. Disruptions to the BBB and normal immune processes amongst the aged population could also result in increased entry of HSV-I and influenza A into the CNS during infection/HSV-I reactivation. Since HSV-I and some strains of influenza A have demonstrated their ability to infect the CNS (especially amongst the elderly) without immediately fatal consequences, the effects of these viral infections in host neurones in the CNS will be reviewed, with a focus on PD-related pathology.

## Viral infection in the CNS: inflammation, synaptic dysfunction, and autophagy disruption

Upon viral infection, the host immune system usually becomes activated and attempts to remove or destroy the invading pathogen via inflammatory mediators, autophagy degradation, or sometimes controlled cell death of infected cells [81]. Although viral pathogens have evolved multiple ways of evading the host immune response (and so host clearance of viral pathogens), host immune circumvention is dependent on virus strain, evasion strategy, and host cell type. Of relevance to PD, the viral evasion of the host immune response may be modulated by BBB integrity, CNS immune cell sensitivity, and duration/severity of infection. The next few sections review viral modulation of the host immune/autophagy response due to HSV-I or influenza A infection. Virus mediated inflammation, synaptic dysfunction, and autophagy disruption in the CNS will be discussed.

### Neuroinflammation

The human immune system is divided into the adaptive (memory-based specific response) and innate (genetically conserved, non-specific response) immune systems [82]. Macrophages are able to attack pathogens due to pattern-recognition receptors (PRRs) that have evolved to recognize pathogen-associated molecular patterns (PAMPs) [83,84]. Meanwhile, the adaptive immune response uses lymphocytes (B and T cells) to ‘remember’ and attack the pathogen more efficiently [85,86]. This immune response sometimes includes cytotoxic lymphocytes, which kill and destroy infected host cells.

Of importance to HSV-I, cluster of differentiation 8 (CD8^+^) T cells have been found to have HSV-I epitopes and block reactivation [87,88]. Although involved in hindering HSV-I reactivation, there are suggestions that these T cells lead to chronic inflammation. Residual lymphocytes were found to recognize HSV-I during latency in the TG, resulting in cytokine release, T-cell exhaustion, and eventual allowing of viral reactivation [89–91]. The H5N1 influenza A strain was also found to induce excessive peripheral T-cell activation [92]. There is evidence of T-cell population modulation in PD as well. T-cell population increase/decrease and impairment in PD depends on T-cell type, and more recently T cells have been found to recognize α-synuclein epitopes [93,94]. Interestingly, recent studies identified homologous cross-reactivity between HSV-I and α-synuclein, suggesting that HSV-I may induce an autoimmune response [95]. Indeed, auto-antibodies against HSV-I peptide were cross-reactive with an α-synuclein epitope [95].

While lymphocytes are involved in the adaptive immune response, the innate immune system also initiates an immediate response due to PAMPs. A key set of PRRs regulating the innate immune system are the Toll-like receptors (TLRs). TLRs are glycoprotein transmembrane receptors that recognize PAMPs (such as lipopolysaccharides, dsDNA/RNA, ssRNA) [96]. Of significance to HSV-I and influenza A, TLR3 is known to recognize viral dsRNA that is present during the viral life cycle within infected host cells [97,98]. TLR3 activation leads to pro-inflammatory cytokine and type I IFN-α/β production, and regulation of DNA expression through nuclear factor κ-light-chain-enhancer of activated B (NF-κB) and IFN regulatory factor (IRF) activation [99].

Neuroinflammation in PD patients has previously been investigated to characterize potential biomarkers. Genetic mutations in PD-related genes (*lrrk2* and *parkin*) have been found to regulate the immune system response [100–104]. Also, single nucleotide polymorphisms in the MHC-II (an antigen-presenting component of specific adaptive immune cells) locus were associated with an increased incidence of PD [105–108]. *Post-mortem* studies have found increased levels of pro-inflammatory cytokines [109]. They also found increased levels of IFNs and p65 subunits of NF-κB [110]. Cerebrospinal fluid (CSF) and peripheral levels of cytokines are also elevated in PD [110,111]. Although the role of these cytokines/IFNs in PD is unknown, animal models have demonstrated that increases in pro-inflammatory mediators results in dopaminergic neurodegeneration [112,113]. Examination of the TLR profile in animal models found increases in TLR3/4 expression in the striatum in response to OS and the pesticide rotenone, possibly leaving these cell populations/brain regions more sensitive to an infection [114]. Interestingly, certain viral infections have found ways to circumvent IFN-stimulated pathways, possibly allowing them to enter and remain dormant in the CNS.

Although the host immune system is well evolved to combat viral infections through type 1 IFNs and IFN-stimulated genes (ISGs), HSV-I and influenza A have also evolved ways to evade this host immune response. HSV-I proteins inhibit NF-κB and IRF3 (a TLR3 downstream regulator of IFNs) activation [115–117]. The influenza A non-structural protein 1 (NS1) prevents the host innate immune response and cellular apoptosis of infected cells by suppressing IFN activation through multiple routes [118]. Also, NS1 regulates IFN-α/β receptor subunit 1 (IFNAR1) surface expression [119]. Due to suppression of the innate immune system, influenza A infection of neurones only leads to increases in tumor necrosis factor-α (TNF-α) release, not interleukin-6 (IL-6) or IFNs [120].

Despite multiple HSV-I proteins working to dampen IFN signaling, still there has been clear evidence of IFN signaling and regulation of viral replication in HSV-I infected cells. This is not surprising since HSV-I inhibition or activation of IRF3 appears to be cell-type dependent [117]. Sensory neurones, where HSV-I latency is generally maintained, are innately unable to mount a large IFN response [121,122 ,123]. This may be why sensory neurones are ideal for HSV-I to maintain latency, but even so, some level of IFN signaling may be required for HSV-I reactivation. Latency-associated transcripts (LATs) have not been found to produce an inflammatory cytokine/IFN response themselves, but instead may require some cytokines/IFNs to initiate reactivation [124,125]. One study suggested that IFNs regulate LAT expression in a way that benefits HSV-I infection; by promoting neurone cell survival throughout latency, HSV-I is provided an opportunity for reactivation and viral spread [125]. Interestingly, neuronal IFN-β suppression was associated with α-synuclein accumulation and PD-like neurodegeneration [126].

Although HSV-I and influenza A viruses have evolved ways to circumvent neuronal innate immune sensing of infection, other CNS cells can still sense and defend against pathogens (see Table 2). Glial cells in the CNS mainly function as regulators of the cellular environment to promote healthy neuronal cell function. Astrocytes (the most abundant cell type in the CNS) support neurone homeostasis by regulating synaptic activity, assisting in BBB formation, and interacting with immune cells. They regulate neurotransmission and metabolism by controlling extracellular potassium levels, uptake of neurotransmitters (such as glutamate), and storing glycogen/exporting lactate [127]. Microglia cells act as resident immune cells in the CNS, with the capability of sensing, engulfing, and degrading invading pathogens [128]. The activation of microglia can have neuroprotective or neurotoxic effects depending on their microenvironment. When activated, some microglia release reactive oxygen species (ROS), inducible nitric oxide synthase (iNOS), and cytokines [129]. These oxidative species and cytokines interact with dopaminergic neurones to regulate cell fate during stress [129].

**Table 2:** Viral induced molecular/cellular changes related to PD pathology

Theme	Condition		
	PD	HSV-I	Influenza A
***Inflammation***			
*Glial cells*	Activated microglia and astrogliosis in PD midbrain [130,131]	↑ microglia in HSV-I infected ependymal [132]	H1N1 ↑ astrogliosis and activated microglia in SN and VTA [133]
*T cells*	T-cell modulation and recognition of α-synuclein epitopes in PD [93,134]	Exhausted T cells in HSV-I infected brain stem and TG express HSV-I epitope [87,89,132]	H5N1 causes excessive peripheral T-cell activation [92]
*Cytokines*	↑ CSF and peripheral cytokines in PD [110,111]	T-cell associated cytokines ↑ in HSV-infected TG [88,90,91]	H5N1 ↑ astrocyte/neuronal cytokines [135,136]
***Autophagy***			
*Disruption*	Autophagic and lysosomal defects in PD neurones [137,138]	PKR inhibition disrupts autophagy/autophagosome formation [139,140,141]	PKR inhibition and autophagosome/lysosome fusion blocked [142,143]
***Synapse***			
*Proteins*	Redistribution of synaptic proteins in PD models [144,145]	↓ synapsin-1 and synaptophysin in murine cortical neurones [146,147]	H5N1 inhibits PSD-95; SNAP25 differentially expressed in neonatal infection [148,149]
*Activity*	↓ synaptic connectivity and glutamatergic synapse loss in PD models [150,151]	Reduced NMDAR and synaptic activity in HSE patients [146,152]	↓ neuronal excitatory synaptic activity and amplitude [153]

As detailed above, multiple parallels can be drawn between PD pathology and the potential molecular and cellular consequences of HSV-I/influenza A infection. Abbreviations: NMDAR, N-methyl-d-aspartate receptor; PKR, protein kinase R; PSD-95, post-synaptic density protein-95; SNAP25, synaptosomal-associated protein 25; VTA, ventral tegmental area.

Astrocytes and microglia participate in the defense against viral spread throughout the CNS [154]. Although HSV-I may find a safe haven in sensory neurones, replication in these neurones for reactivation may alert neighboring astrocytes. These cells recognize extracellular dsRNA since they can express cell surface TLR3 [155]. Indeed, previous studies have found astrocytes to be reactive to a synthetic mimetic of dsRNA, albeit with conflicting conclusions [155–160]. One study found synthetic dsRNA to produce an anti-inflammatory response in astrocytes [156], while others found a pro-inflammatory response [157,160]. The reasons for these differences may be due to astrocyte source (fetal or adult). Overall, synthetic dsRNA treatment in human astrocytes *in vitro* was found to cause increases in IFNs, IL-6, and a down-regulation in connexin 43 (a crucial protein for intercellular gap junctions between astrocytes and maintaining BBB integrity) [157,160,161]. Interestingly, a rat study also found synthetic dsRNA to attenuate astrocytic l-glutamate uptake by inhibiting EAAT1/GLAST transporter gene transcription [162]. Studies examining HSV-I infection in the mouse CNS found increased inflammation and ROS [163,164]. These studies suggest viral infection and replication in neurones near astrocytes could cause an inflammatory response and disrupt healthy astrocyte function, possibly leading to neuronal signaling dysfunction and cell death.

Of relevance to HSV-I and glia activity, a study describing a mouse model of HSE found lytic genes (*ICP0* and *ICP27*) to sustain their expression long into ‘latency’ within the brain ependymal after HSE recovery [132]. This HSV-I gene expression profile differs from its life in the TG and was associated with a loss of effector T-cell function and an increase in microglia in the region. Although most humans infected with HSV-I never experience an episode of HSE during their lifetime, the present study not only further demonstrates that not all cell types respond in the same way to HSV-I, but that HSV-I can infect regions of the CNS without lethal consequences.

### Synaptic dysfunction

Previous models of PD have suggested that synaptic dysfunction (such as alterations to long-term potentiation/depression, changes in synaptic proteins, and N-methyl-d-aspartate receptor (NMDAR) subunit composition) in nigrostriatal and corticostriatal pathways could be responsible for the physical manifestations of DA loss in the SN [165–167]. *Post-mortem* studies have found decreases in glutamatergic synapses and α-amino-3-hydroxy-5-methyl-4-isoxazolepropionic acid (AMPA) GluR1 in the striatal regions of PD patients [168,150]. Also, human-induced pluripotent stem cell derived neurones from familial PD patients demonstrated reduced synaptic connectivity and hindered neurite outgrowth [151]. Although more research needs to be conducted to better understand synaptic dysfunction in PD, HSV-I and influenza A associated modifications to normal synaptic function are worth review (see Table 2).

Influenza A infection has been found to disrupt synaptic activity through modulation of host gene expression and interaction with synaptic related proteins. Pandemic and seasonal influenza A infections were examined for their modulation of genes in the CNS [169]. Pandemic influenza strains were associated with down-regulation of ‘neuron projection,’ ‘synapse assembly,’ and ‘calcium channel activity’ related genes [169]. Genes that were down-regulated compared with the seasonal flu strain included glycoprotein M6A, protocadherin α-subfamily C2, and cAMP-regulated phosphoprotein. Influenza A NS1 and nucleoprotein (NP) were also found to modify the host synapse. The influenza A NP was found to localize within dendritic spine-like structures of hippocampal neurones, resulting in reduced spontaneous excitatory synaptic frequency and decreased amplitude of excitatory post-synaptic currents [153]. Also in hippocampal neurones, the post-synaptic density protein-95 (PSD-95)/discs-large/ZO-1 (PDZ) motif of the C-terminus of H5N1 influenza A NS1 (not H1N1 influenza A NS1) was found to bind to PSD-95 [148]. NS1 binding to PSD-95 was suggested to prevent normal post-synaptic processes.

Although there is no evidence of direct inhibition of synaptic proteins, HSV-I infection is associated with changes in the host synapse. HSE patients are often found to have NMDAR antibodies, with a reduction in NMDAR and synapsin protein in murine hippocampal neurones after treatment with HSE patient serum [146]. In an animal model, HSV-I infection of murine cortical neurones resulted in reduction in synapsin-1 and synaptophysin proteins, and disrupted synaptic transmission [147]. Although synaptic dysfunction in PD may be a result of other features of PD pathology (such as α-synuclein aggregation or DA striatal denervation), it is worth noting viral induced changes in synaptic function. HSV-I or influenza A infection may exacerbate already stressed synaptic connections or weaken synaptic activity before other PD pathological features have fully manifested.

### Autophagy

The autophagy process is fundamental for cellular homeostasis. Briefly, unwanted components (misfolded proteins, foreign structures, dysfunctional proteins etc.) are engulfed in double-membraned vesicles (autophagosomes) for digestion and substrate recycling [170]. Autophagy pathways have been suggested to be very important for amounting an antiviral defense in non-replicating cells [171]. Epithelial cells infected with HSV-I can produce pro-inflammatory cytokines and undergo cell death to prevent viral spread without permanent tissue damage because they can be replaced afterward. Non-replicating cells, such as neurones, may be more reliant on autophagy processes to limit viral replication and viral spread without undergoing cell death [172]. Similar to IFN signaling evasion, HSV-I and influenza A proteins have evolved ways to disrupt autophagy events to prevent clearance of viral components from host cells during latency and replication.

Previous studies have identified ICP34.5 as a crucial HSV-I protein for inhibiting autophagic degradation of virion structures [173,139]. Multiple HSV-I and influenza A proteins are able to disrupt autophagy. HSV-I ICP34.5 is able to indirectly inhibit protein kinase RNA-activated (PKR), while HSV-I US11 directly binds to PKR to prevent activation [174–176]. Influenza A NS1 and NP also inhibit PKR [142]. This PKR activation inhibition prevents PKR-mediated autophagy activation [139,142]. Also, HSV-I ICP34.5 has been found to bind directly to Beclin-1 [140]. Beclin-1 binds with other autophagy components to promote the formation of autophagosomes [141]. The amino acid region 68–87 of ICP34.5 binds to Beclin-1, leaving the section that functions to inhibit PKR signaling to remain open [140]. HSV-I ICP34.5 inhibition of autophagy through Beclin-1 binding also prevents antigen presentation and cluster of differentiation 4 (CD4^+^) T-cell response [177]. Further investigation into the consequences of ICP34.5-mediated autophagy disruption needs to be done to understand the effects on host neurone homeostasis beyond increased neurovirulence of HSV-I.

Viral mediated autophagy disruption or suppression could lead to a decrease in clearance of misfolded/aggregated proteins. Studies examining *post-mortem* AD brains found HSV-I DNA to be associated with amyloid-β plaques [70,22,178]. Although a majority of HSV-I DNA positive CNS neurones were found to have amyloid-β plaques, there was no correlation between amyloid-β plaque containing neurones and presence of HSV-I DNA [70]. These findings suggest that HSV-I infection may cause an increase in amyloid protein aggregation. Further investigation should be conducted to determine if there may also be an association between HSV-I DNA positive neurones and α-synuclein aggregation. A study by Santana et al. [179] found HSV-I infection in neuronal cell cultures to cause an increase in amyloid-β aggregation accumulation, along with an increase in microtubule-associated protein 1A/1B-light chain (LC3-II). This study further supports the theory that HSV-I may contribute to amyloid protein aggregation, but does not refute the possibility that this is related to HSV-I mediated autophagy disruption since ICP34.5 disrupts autophagy through Beclin-1 binding, not LC3-II. Also, the lack of correlation between amyloid-β plaque containing neurones and HSV-I DNA positivity may be due to cross-seeding. HSV-I may cause autophagy disruption in innervating neurones (possibly latent sensory neurones), leading to amyloid fibril accumulations that may have the capability of spreading to other non-HSV-I DNA positive neurones. These protein aggregates may then be transported to innervating neurones or be exocytosed for extracellular cross-seeding. In general, autophagy disruption has been previously found to cause an increase in neurodegeneration, presynaptic α-synuclein accumulation, neuronal inclusions, and dopaminergic axon and dendritic degeneration [137,180].

## Conclusion

**Figure 1:**
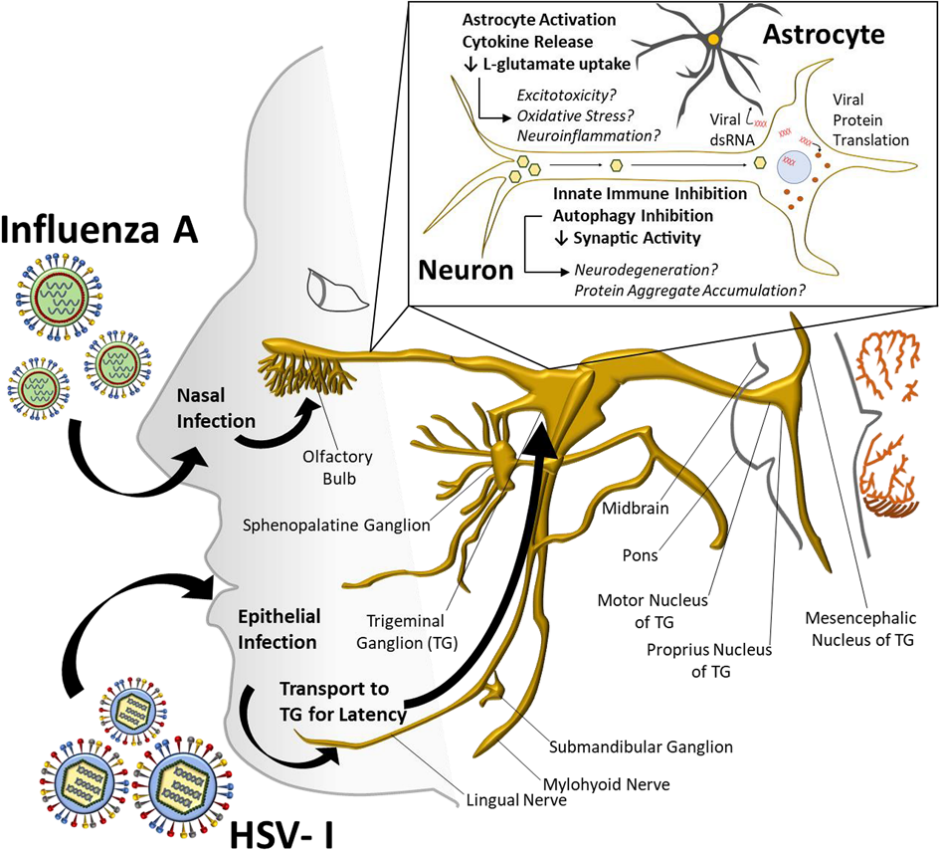
HSV-I and Influenza A viral infections may lead to PD-like pathology HSV-I and influenza A viral infections have the potential to cause molecular and cellular changes that can alter healthy neuron function within the CNS. Viral transcripts/proteins due to HSV-I/influenza A infection may cause inflammation, autophagy disruption, and synapse dysfunction, possibly contributing to a PD-like pathology.

The purpose of this review was to demonstrate the need to revisit the association between viral infections and PD. It is clear that parallels can be drawn between viral-induced changes in the CNS (ranging from chronic inflammation to synaptic dysfunction) and PD pathology (Figure 1). Further investigation of viral infections (specifically HSV-I and influenza A) should be conducted to determine if intervention can suppress the long-term consequences in the CNS and possibly mitigate the association between viral infections and incidence of PD.

## Abbreviations

AD: Alzheimer’s disease
BBB: blood–brain barrier
CNS: central nervous system
DA: dopamine
EL: encephalitis lethargica
HCV: hepatitis C virus
HSE: herpes simplex encephalitis
HSV: herpes simplex virus
IFN: interferon
IL-6: interleukin-6
iPD: idiopathic Parkinson’s disease
IRF: IFN regulatory factor
LAT: latency-associated transcript
LC3-II: microtubule-associated protein 1A/1B-light chain 3
NF-κB: nuclear factor κ-light-chain-enhancer of activated B cell
NMDAR: N-methyl-d-aspartate receptor
NP: nucleoprotein
NS1: non-structural protein 1
OS: oxidative stress
PAMP: pathogen-associated molecular pattern
PD: Parkinson’s disease
PKR: protein kinase R
PRR: pathogen-recognition receptor
PSD-95: post-synaptic density protein-95
ROS: reactive oxygen species
SN: substantia nigra pars compacta
TG: trigeminal ganglia
TLR: toll-like receptor
